# p70S6 kinase regulates oligodendrocyte differentiation and is active in remyelinating lesions

**DOI:** 10.1093/braincomms/fcac025

**Published:** 2022-02-12

**Authors:** Karelle Benardais, Isis M. Ornelas, Melissa Fauveau, Tanya L. Brown, Lisbet T. Finseth, Radmila Panic, Cyrille Deboux, Wendy B. Macklin, Teresa L. Wood, Brahim Nait-Oumesmar

**Affiliations:** 1 Sorbonne Université, Institut du Cerveau - Paris Brain Institute - ICM, Inserm, CNRS, APHP, Hôpital de la Pitié-Salpêtrière, Paris, France; 2 Department of Pharmacology, Physiology & Neuroscience, New Jersey Medical School, Rutgers University, Newark, NJ 07101, USA; 3 Department of Cell and Developmental Biology, University of Colorado School of Medicine, Aurora, CO 80045, USA

**Keywords:** p70S6K1, pS6RP, oligodendrocyte, (re)-myelination, multiple sclerosis

## Abstract

The p70 ribosomal S6 kinases (p70 ribosomal S6 kinase 1 and p70 ribosomal S6 kinase 2) are downstream targets of the mechanistic target of rapamycin signalling pathway. p70 ribosomal S6 kinase 1 specifically has demonstrated functions in regulating cell size in *Drosophila* and in insulin-sensitive cell populations in mammals. Prior studies demonstrated that the mechanistic target of the rapamycin pathway promotes oligodendrocyte differentiation and developmental myelination; however, how the immediate downstream targets of mechanistic target of rapamycin regulate these processes has not been elucidated. Here, we tested the hypothesis that p70 ribosomal S6 kinase 1 regulates oligodendrocyte differentiation during developmental myelination and remyelination processes in the CNS. We demonstrate that p70 ribosomal S6 kinase activity peaks in oligodendrocyte lineage cells at the time when they transition to myelinating oligodendrocytes during developmental myelination in the mouse spinal cord. We further show p70 ribosomal S6 kinase activity in differentiating oligodendrocytes in acute demyelinating lesions induced by lysophosphatidylcholine injection or by experimental autoimmune encephalomyelitis in mice. In demyelinated lesions, the expression of the p70 ribosomal S6 kinase target, phosphorylated S6 ribosomal protein, was transient and highest in maturing oligodendrocytes. Interestingly, we also identified p70 ribosomal S6 kinase activity in oligodendrocyte lineage cells in active multiple sclerosis lesions. Consistent with its predicted function in promoting oligodendrocyte differentiation, we demonstrate that specifically inhibiting p70 ribosomal S6 kinase 1 in cultured oligodendrocyte precursor cells significantly impairs cell lineage progression and expression of myelin basic protein. Finally, we used zebrafish to show *in vivo* that inhibiting p70 ribosomal S6 kinase 1 function in oligodendroglial cells reduces their differentiation and the number of myelin internodes produced. These data reveal an essential function of p70 ribosomal S6 kinase 1 in promoting oligodendrocyte differentiation during development and remyelination across multiple species.

## Teaser

p70 S6 kinase (S6K) activity in oligodendroglia corresponds to developmental myelination and remyelination in rodents and is present in active lesions of multiple sclerosis. S6K1 promotes the expression of the myelin basic protein in rodent oligodendrocyte precursor cells and regulates the number of myelin internodes in zebrafish.

## Graphical Abstract

**Graphical Abstract:**
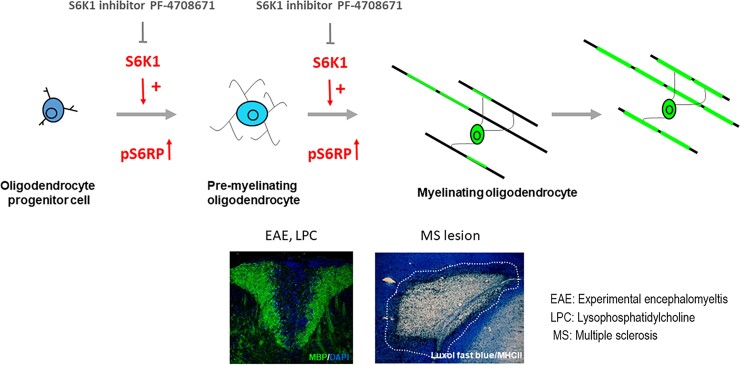

## Introduction

The p70 ribosomal S6 kinase 1 (S6K1) is a downstream target of signalling pathways with known roles in cell growth.^1^ Deletion of S6K in *Drosophila* causes a developmental delay and reduced body size due to a general reduction in cell size.^2^ In mammals, two S6K family members arise from distinct genes, S6K1 and S6K2. S6K1 is the major regulator of cell size, particularly in nutrient- and insulin/insulin-like growth factor-sensitive tissues. Consistent with the phenotype in flies, S6K1 deletion in mice results in a 20% reduction in body weight due to a large part of a reduction in cell size, particularly in the pancreas (beta cells), skeletal muscle and adipose tissues.^1,3^ S6K1-deficient mice are also hypo-insulinaemic due to compromised pancreatic beta-cell growth. Mice with a deletion of S6K2 have very little phenotype and normal body size; however, the double knockouts (KOs) of S6K1/2 have a more severe phenotype than the S6K1 KO mice and die perinatally,^4^ suggesting some compensation by the alternate kinase in the individual KOs.

The S6 ribosomal protein (S6RP), a component of the 40S ribosomal subunit, is a primary target of the S6Ks. Although the S6Ks have long been considered regulators of protein translation due to their phosphorylation of S6RP (pS6RP), as well as other targets in the protein synthesis machinery, recent studies revealed that the S6Ks neither regulate global translation nor translate specific mRNA classes.^4,5^ Rather, the combined deletion of S6K1/2 has a dramatic effect on the transcriptional programme for ribosome biogenesis without altering translation initiation.^5^ Thus, the phenotypes from the mouse KOs reveal both distinct and overlapping functions for the two S6K family members with S6K1 having a prominent role in cell size in specific tissues, whereas both members regulate ribosome biogenesis.

One of the major pathways identified upstream of the S6Ks is the mammalian target of rapamycin (mTOR), a serine/threonine protein kinase that regulates many aspects of cell growth, proliferation and differentiation. In initial studies in oligodendroglia, we and others identified mTOR as a downstream mediator of the PI3K/Akt signalling pathway during oligodendrocyte differentiation and myelination both *in vitro* and *in vivo*.^6–11^ mTOR forms two complexes, mTORC1 and mTORC2, defined by the specific regulatory proteins with which mTOR interacts including raptor in mTORC1 and rictor in mTORC2, among others.^12^ mTORC1 is the complex responsible for S6K phosphorylation and activation, and loss of raptor in oligodendrocyte progenitor cells (OPCs) phenocopies the mTOR conditional KO.^13^ Taken together, these data support the hypothesis that mTOR signalling through mTORC1 is critical for normal oligodendrocyte differentiation and myelination in the developing CNS.

The goal of the present studies was to determine the activity of S6K during oligodendrocyte differentiation, myelination and remyelination in the CNS. Our data support a prominent role for S6K in oligodendrocyte lineage cells in the transition from a precursor to a mature oligodendrocyte at the onset of myelination during development in the mouse, as well as in remyelination in the adult CNS in mouse demyelinating models and active multiple sclerosis (MS) lesions. We also provide compelling evidence indicating that S6K1 regulates OPC differentiation during developmental myelination in both rodents and zebrafish.

## Material and methods

### Mouse developmental analysis

For developmental analyses of pS6RP expression, C57BL/6J wild-type mice at postnatal day (PND) 0, 4, 7, 10, 21 and 30 (*n* = 3 per timepoint) were perfused with 2% paraformaldehyde (PFA) after lethal anaesthesia with ketamine (100 mg/kg)/Xylazine (10 mg/kg). Spinal cords were dissected and post-fixed in the fixative solution for 1 h at 4°C, cryopreserved in a gradient of 10% and then 20% sucrose overnight at 4°C and embedded in Tissue-Tek® O.C.T Compound (Thermo Fisher Scientific). Twelve-micrometre serial coronal sections were performed on a cryostat (Leica, Germany), and slides were stored at −20°C until use for immunohistochemistry (IHC). All experimental procedures were reviewed and approved by the Charles Darwin Ethical Committee (Agreement no. 05248.02) and followed EU regulations on animal handling and use for research purposes.

### Lysophosphatidylcholine focal demyelination

Ten-week-old C57BL/6J mice (*n* = 6 per timepoint) were used for focal demyelinated lesions in dorsal white matter spinal cords by stereotaxic injections of 1% lysophosphatidylcholine (LPC; Sigma-Aldrich), as previously described with slight modifications.^14^ Briefly, the animals were anaesthetized by isoflurane, shaved and placed on the stereotactic frame. During the procedure, the animal temperature was monitored and maintained at 37°C. A 3 cm midline incision was made at the thoracic levels (T11–T13) vertebra. Spinal cord dura was removed with a 32G needle. Using a micromanipulator and a diffusion pump linked to a Hamilton syringe, 1 µl of either 1% LPC in saline solution or saline solution alone (used as a control) was injected into the dorsal funiculus of the spinal cord at a speed of 0.3 µl/min. The capillary tube was left in place for 5 min to prevent backflow of solution. Muscle tissue and skin overlying the spinal cord dorsal column were sutured. Post-operative analgesics (carprofen, 5 mg/kg subcutaneously) were given to the mice every 12 h for 2 days.

### Myelin oligodendrocyte glycoprotein-induced experimental autoimmune encephalomyelitis

Twelve week-old C57BL/6J female mice (Elevage Janvier, France) were immunized subcutaneously, as previously described,^15^ with an emulsion consisting of 200 µg of synthetic myelin oligodendrocyte glycoprotein (MOG)_35–55_ peptides (NeoMPS) in complete Freund's adjuvant (CFA, Difco), supplemented with 500 µg of heat-inactivated *Mycobacterium tuberculosis* (strain H37Ra, Difco). Mice (*n* = 25) were injected retro-orbitally with Pertussis toxin (List Biological Laboratories) on the day of the immunization and 48 h later. Animals were scored daily for clinical signs (0: healthy; 0.5: limp tail; 1: tail paralysis; 2: hindlimb paresis; 2.5: one hindlimb paralysis; 3: both hindlimb paralysis; 3.5: forelimb weakness; 4: forelimbs paralysis; 5: death). All experimental procedures were reviewed and approved by the Charles Darwin Ethical Committee (Agreement no. 05248.02) and followed EU regulations on animal handling and use for research purposes.

### Tissue processing

Animals were deeply anaesthetized with an intraperitoneal injection of pentobarbital sodium (30 mg/kg) and perfused through the left ventricle of the heart with 2% PFA in phosphate buffer saline (PBS). Spinal cords were carefully dissected, post-fixed in the same solution overnight and cryoprotected in 10% and 30% sucrose in PBS for 24 h each. The resulting tissue was frozen and used to obtain 12-μm cryostat coronal sections.

### MS post-mortem tissue

To examine the expression of pS6RP, snap-frozen post-mortem brain samples from MS patients and control patients were obtained from the UK MS tissue bank (Imperial College, London, approved by the Wales Research Ethics Committee, ref. no. 18/WA/0238). Eight MS and three control cerebellar tissue blocks were used in this study (Supplementary Table 1). Twelve-micrometre frozen sections were performed on a cryostat and processed as previously described.^16^ Histological characterization of MS lesions was performed using Luxol fast blue/major histocompatibility complex class II (MHCII) staining and lesions were classified according to histological criteria,^17,18^ as active (*n* = 3), chronic active (*n* = 1), chronic inactive (*n* = 3) and remyelinated (shadow plaques, *n* = 2). Prior to immunofluorescence histochemistry, sections were treated 5 min with an antigen retrieval solution (Vector unmasking solution) according to the manufacturer's instructions. Immunofluorescence labelling was performed as described for the mouse experiments.

### Immunohistochemistry

We analysed mTOR signalling using an antibody against pS6RP in the corpus callosum and spinal cord of the control mice, as well as in LPC-induced demyelinated and MOG-induced experimental autoimmune encephalomyelitis (EAE) lesions. For IHC, sections were incubated with 4% BSA (bovine serum albumin), 0.5% Triton X-100 for 45 min at room temperature (RT) and then with primary antibodies overnight at 4°C. Sections were rinsed 3 times with PBS/0.1% Triton X-100 and then incubated with the appropriate secondary antibodies, as previously described.^19^ Sections were counterstained with 4′,6-diamidino-2-phenylindole (DAPI) and mounted with Fluoromount (CliniSciences, France). Primary antibodies were rat anti-platelet-derived growth factor receptor alpha (PDGFRα) (1:100; BD Bioscience), mouse anti-adenomatous polyposis coli (APC) (1:100; clone CC1, Calbiochem), mouse anti-breast carcinoma amplified sequence 1 (BCAS1) (1:100; NaBC1, Santa Cruz), goat anti-Sox10 (1:50; R&D System), mouse anti-MOG (1:2; clone C18C5, kindly provided by C. Linnington, University of Glasgow, Glasgow, UK), rat anti-myelin basic protein (MBP) (1:200; Abcam), mouse anti-glial fibrillary acidic protein (GFAP) (1:100; Millipore), mouse anti-CD45 (1:100; Invitrogen), mouse anti-human leucocyte antigen (HLA)-DP, DQ, DR Antigen (1:100; clone CR3/43, Dako), rabbit anti-pS6RP (Ser235/236) (1/100; Cell Signaling, #4858) and rabbit anti-pS6RP (Ser 240/244) (1:1000; Cell Signaling, #5364). Secondary antibodies (all from Thermo-Fischer) were donkey anti-goat Alexa568- or Alexa647-conjugated IgG, anti-mouse Alexa488 or Alexa568-conjugated IgG2b, rat donkey anti-goat Alexa647-conjugated IgG and donkey anti-rabbit Alexa488-conjugated IgG. Alexa-conjugated secondary antibodies were used at 1:1000 dilution.

### Oligodendroglial cell cultures and immunocytochemistry

Primary rat OPCs were purified from cortical mixed glial cultures prepared from PNDs 0–2 Sprague–Dawley rat pups as previously described.^9,20^ Purified OPCs were seeded onto poly-d-lysine-coated T75 flasks at a density of 2 × 10^4^ cells/cm^2^ in a chemically defined medium (N2S) consisting of 66% N2B2 (DMEM/F12 supplemented with 0.66 mg/ml BSA, 10 ng/ml d-biotin, 5 µg/ml insulin, 20 nM progesterone, 100 µM putrescine, 5 ng/ml selenium, 50 µg/ml apo-transferrin, 100 U/ml penicillin, 100 µg/ml streptomycin) supplemented with 34% B104 conditioned media, 5 ng/ml fibroblast growth factor and 0.5% foetal bovine serum. Purified OPCs were amplified in N2S, passaged once with papain and induced to differentiate using an established mitogen withdrawal protocol in N2B2 media with 30 ng/ml triiodothyronine (T3) and in the presence or absence of the mTOR inhibitor, rapamycin (15 µM) as recently described^6^ or of the S6K1 inhibitor, PF-4708671 (1 or 10 µM). Control cultures received vehicle alone (DMSO). N2B2 + T3 differentiation media with or without drug treatments was replenished every 48 h during experiments. Rat (CG)-4 cells, an OPC line that gives rise to astrocytes and oligodendrocytes,^21^ were cultured and differentiated as for the primary rat OPCs.

For immunolabelling, live CG4 cells were incubated for 1 h at 37°C with the GalC hybridoma (1:50),^22^ rinsed in PBS and then fixed in 2% PFA for 10 min. After rinsing in PBS, cells were then incubated with an antibody against pS6RP (1:200; Cell Signaling #4858) in PBS with 0.02% Triton X-100. Cells were washed again in PBS and incubated in secondary antibodies: anti-Rabbit Alexa 488 (1:2000; Thermo-Fischer) and anti-mouse IgG3-TRITC (1:100; Clinisciences) for 1 h at RT. Cells were washed, stained with DAPI and mounted using a fluoromount media.

For MBP/pS6RP staining of primary cells, cultures were fixed in 3% PFA for 15 min and permeabilized with 0.3% Triton X-100 for 15 min. Cells were blocked in 3% BSA and 3% goat serum in PBS for 1 h and incubated overnight with primary antibodies to MBP (1:500; Abcam ab40390) and pS6RP (1:200; Cell Signaling #4858) in blocking buffer. Cells were washed and incubated for 1 h in secondary antibodies: Alexa Fluor488 goat anti-mouse IgG (1:750; Invitrogen A11001) and Alexa Fluor647 goat anti-rabbit IgG (H + L) (1:750; Invitrogen A21245). Cells were incubated with DAPI 1:5000 for 5 min and then washed and mounted using ProLong Gold mounting media. Images were acquired using an Olympus Provis AX70 microscope.

### Western blot

Protein isolation and western blot protocols were performed as described recently.^6^ Following treatments, cells were washed twice with ice-cold PBS and total cell lysates were harvested in RIPA lysis buffer (Pierce) with 1/50 protease inhibitor cocktail (Sigma, St. Louis, MO, USA). The lysates were briefly sonicated and stored at −80°C prior to western analysis. The RC-DC protein assay (BioRad) was performed to determine protein concentration. About 20 µg of the total protein per sample was boiled for 5 min and separated by SDS–PAGE on Bis–Tris mini-gels (Invitrogen). Separated proteins were then transferred to nitrocellulose membranes and blocked in 5% milk/TBS-0.1% Tween for 1 h at RT. Membranes were incubated in the presence of primary antibodies against MBP (Biolegend 808402; 1:1000), Actin (Sigma A5441; 1:10 000), pS6RP (Cell Signaling 4858; 1:4000) or total S6RP (Cell Signaling 2217; 1:1000) diluted in 5% BSA/TBS-0.1% Tween overnight at 4°C. The following day, membranes were washed 3 times for 10 min with TBS-0.1% Tween and incubated for 1 h at RT in 5% milk/TBS-0.1% Tween containing goat anti-rabbit or goat anti-mouse secondary antibodies at a dilution of 1:5000. The detection of horseradish peroxidase-conjugated secondary antibodies was performed by enhanced chemiluminescence using the Ultra-LUM imaging device. Protein expression levels were quantified using Image J.

### Zebrafish methods

#### Cloning

The Tol2kit was used to construct plasmids.^23^ pEXPR-myrf:S6K1-KR constructs were generated by subcloning the S6K1 cDNA into the middle entry plasmid. This middle entry plasmid was then used for recombination with p5E-myrf, p3E-2AnlsmCherry and pDEST-nonet plasmids, using the Tol2 kit. The resulting plasmids were verified by restriction digest and sequencing. For cloning, we used the following plasmids: P5E-myrf (a gift from Bruce Appel), PRK7-HA-S6K1-KR (a gift from John Blenis, Addgene plasmid # 8985),^24^ pDEST-cmlc2:RFP, pDEST-no heart marker, pEXPR-mbp:mEGFP, pME-, pME-S6K1-KR and p3E-2AnlsmCherry.

#### DNA microinjections

For mosaic labelling, zebrafish embryos were collected and injected with appropriate DNA constructs at the 1–4 cell stage. Embryos were injected with 1 nl of a solution containing 15–25 ng/µl total plasmid DNA, 20 ng/µl tol2 transposase mRNA and phenol red.

#### Live imaging

All zebrafish experiments were approved by the Institutional Animal Care and Use Committee at the University of Colorado School of Medicine. Embryos were raised at 28.5°C in embryo media (EM) and staged according to hour post-fertilization, days post-fertilization (dpf) and morphological criteria.^25^ Zebrafish were anaesthetized using tricaine (MS-222). Embryos were mounted laterally in 1% low-melt agarose and tricaine and imaged directed above the yolk sac extension on a Leica DM-6000 confocal. Individual myelin internodes were traced and analysed in 3D using IMARIS image analysis software (Bitplane). When needed, image brightness and contrast were increased uniformly in individual images.

Oligodendrocyte migration or differentiation was analysed in the zebrafish dorsal spinal cord using Tg(*olig2:EFGP; sox10:TagRFP*) or Tg(*olig2:EGFP; mbp:rfp*) larva, respectively. Embryos were treated with 1% DMSO (control) or 1% DMSO + PF4708671 (Sigma-Aldrich) in EM at 2 dpf. For dorsal migration, larvae were live imaged at 3 dpf after 24 h of exposure. For differentiation, drug + EM was refreshed at 3 dpf and the larva was live imaged at 4 dpf. Embryos were imaged using the Leica DM-6000 confocal at a 0.45 μM step size and 25× water immersion lens. Images were analysed in 3D using IMARIS (Bitplane). Statistical analysis of one-way ANOVA and Dunnett's multiple comparison was performed using Prism 8 (Graphpad Software, LLC).

### Statistical analysis

For developmental analysis of pS6RP expression in the spinal cord, at least three WT C57Bl6 mice were used at each developmental stage (PND0–PND30). For each developmental stage, the number of pS6RP^+^Sox10^+^ and pS6RP^+^CC1^+^Sox10^+^ cells were counted in the dorsal and ventral white matter of the spinal cord (thoracic level), on at least four non-adjacent sections per animal. Data were expressed as the density of positive cells/mm^2^ and as the percentage of total Sox10^+^ oligodendroglia. One-way ANOVA followed with Tukey multiple comparisons test were used for statistical significances, set as *P < 0.05, **P < 0.01 and ***P < 0.001.

For the analysis of pS6RP-expressing cells in LPC lesions of the spinal cord, the number of pS6RP-expressing cells was quantified in at least *n* = 3 C57BL6 adult mice for each timepoint. Six serial spinal cord sections with a demyelinated lesion per animal were used to evaluate the number of pS6RP^+^ cells at each timepoint. Data were expressed as the average density of pS6RP^+^ cells/mm^2^ and ANOVA followed with Tukey multiple comparison tests was used for statistical analysis.

For primary rat OPC experiments, analyses were performed blinded to the treatment group. An unpaired two-tailed *t*-test was performed for analyses comparing two treatment groups with Welch's correction if variances were unequal, and significance was defined as *P* ≤ 0.05. For comparison of S6K1 inhibitor to control in immunostaining experiments for MBP and PS6RP, nine replicate wells were analysed. For all multi-group comparisons, ANOVA was performed followed by Dunnett's multiple comparisons test.

### Data availability

The raw data that support this study are available from the corresponding authors upon reasonable request.

## Results

### pS6RP expression is timely regulated during oligodendroglial cell development

**Figure 1:**
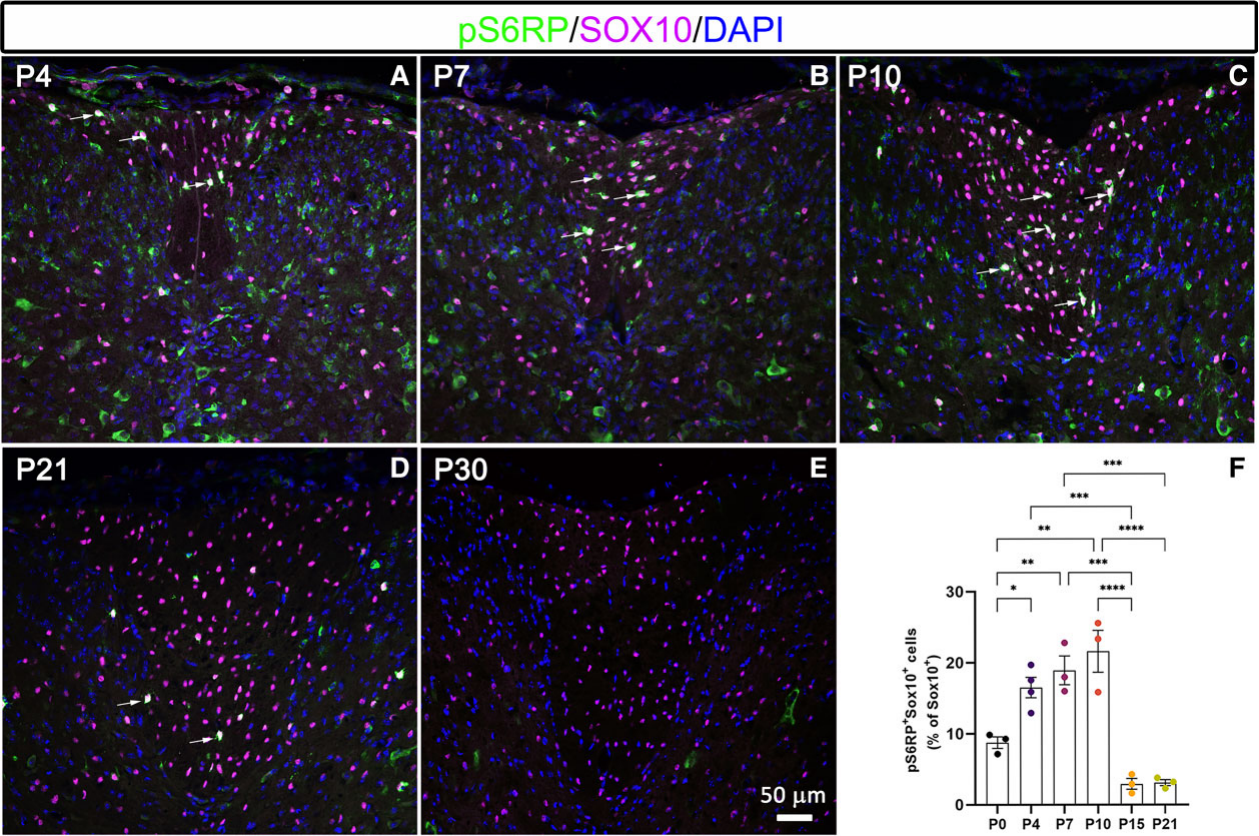
**Developmental expression profile of pS6RP in oligodendroglial cells**. (**A**–**E**) Co-immunolabelling of pS6RP and Sox10 at PND 4 (**A**), PND 7 (**B**), PND 10 (**C**), PND 21 (**D**) and PND 30 (**E**) in the spinal cord. Double-positive cells are indicated by arrows. (**F**) Quantification of pS6RP^+^Sox10^+^ cells shows a peak of pS6RP expression between PND7 and PND10, indicating that pS6RP expression is regulated in oligodendroglial cells during development. Data represent mean ± SEM; *N* = 3 independent experiments for each developmental stage. ANOVA followed with *post hoc* Tukey's pairwise multiple comparison tests: *P < 0.05; **P < 0.01; ***P ≤ 0.001; ****P ≤ 0.0001. Panels (**A**–**E**) are counterstained with DAPI. Scale bar (**A**–**E**): 50 µm.

Our first goal in determining S6K activity in oligodendroglial cells was to define the expression profile for pS6RP (a read-out of S6K enzymatic activity) during oligodendroglial development and myelination. We performed co-immunolabelling for pS6RP and Sox10 in the developing spinal cord of C57BL6 mice (*n* = 3) at PND 0, 4, 7, 10, 15, 21 and 30. Our data revealed pS6RP expression in Sox10^+^ oligodendroglia as early as PND0 in spinal cord white matter. Quantification of pS6RP^+^Sox10^+^ cells indicated that pS6RP expression was upregulated from PND4 to PND10 (Fig. 1A–C and F) in the mouse spinal cord white matter, coinciding with the onset of active myelination. Interestingly, pS6RP protein expression in oligodendroglia peaked at PND10 and then drastically decreased by PND21 (Fig. 1D and F) to levels that were undetectable at PND30 (Fig. 1E), a period corresponding to the completion of spinal cord myelination.^26^ Overall, our data demonstrate that pS6RP is transiently expressed during oligodendrocyte lineage progression and correlates with the onset of OPC differentiation and/or myelination.

### pS6RP is primarily expressed in CC1^+^ maturing oligodendrocytes

**Figure 2:**
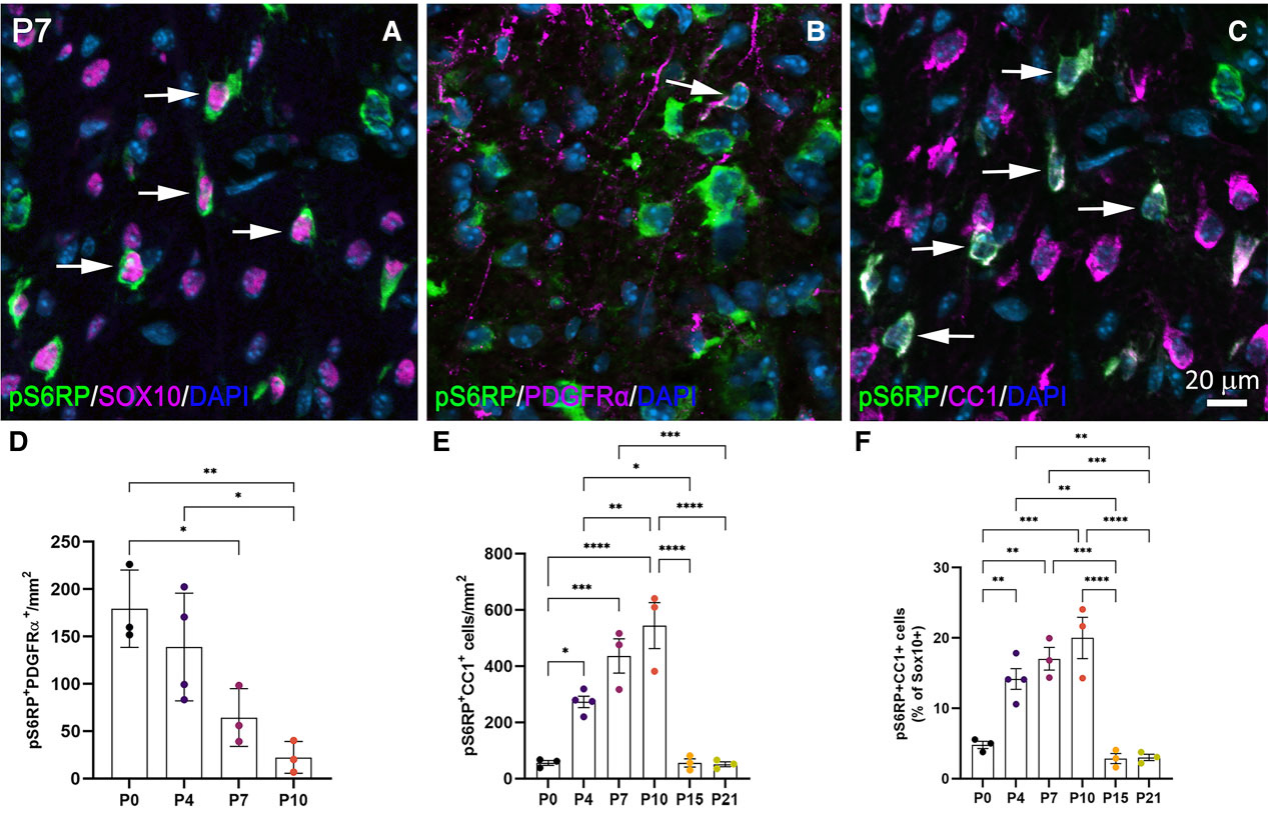
**Characterization of pS6RP expression in oligodendroglial cells**. (**A**–**C**) Co-immunolabelling for pS6RP and Sox10 (**A**), pS6RP and PDGFRα (**B**), pS6RP and CC1 (**C**) in the dorsal funiculus of PND7 WT mice. Double-positive cells are indicated by arrows. (**D**) Number of pS6RP^+^PDGRα^+^ OPCs/mm^2^ from PND0 to PND10. (**E**) The number of CC1^+^ differentiated oligodendrocytes expressing pS6RP from PND0 to PND21. Note that pS6RP is transiently expressed in CC1^+^ differentiated oligodendrocytes. (**F**) Although transient, pS6RP is predominantly located in CC1^+^ differentiated oligodendrocytes between PND4 and PND10. Data represent mean ± SEM; *N* = 3 independent experiments for each developmental stage. ANOVA followed with *post hoc* Tukey's pairwise multiple comparison tests: *P < 0.05; **P < 0.01; ***P ≤ 0.001; ****P ≤ 0.0001. Panels (**A**–**C**) are counterstained with DAPI. Scale bar (**A–C**): 20 µm.

We next assessed whether pS6RP was expressed at specific stages of the oligodendroglial lineage, by performing co-immunolabeling for pS6RP together with Sox10 (Fig. 2A) for all oligodendroglial cells, PDGFRα for OPCs and CC1 (anti-APC) for post-mitotic oligodendrocytes. Our data revealed pS6RP expression in very few PDGFRα^+^ OPCs (Fig. 2B). The number of PDGFRα^+^pS6RP^+^ cells was highest at PND0 (179.18 ± 40.8 PDGFRα^+^pS6RP^+ ^cells/mm^2^) and drastically decreased during the spinal cord development, representing <22.2 ± 16.8 PDGFRα^+^pS6RP^+^ cells/mm^2^ at PND10 (Fig. 2D). pS6RP expression was mainly detected in CC1^+^ oligodendrocytes (Fig. 2C and E). Quantification of pS6RP^+^CC1^+^ cells over the total Sox10^+^ oligodendroglial cell population revealed that pS6RP expression in differentiated oligodendrocytes peaked between PND4 and PND10 in the dorsal funiculus of the spinal cord (Fig. 2F). Nonetheless, quantification of pS6RP^+^CC1^+^ cells among the total CC1^+^ cell population demonstrated that the percentage of differentiated oligodendrocytes expressing pS6RP (∼15–20%) was stable from PND4 to PND10 (data not shown), indicating that pS6RP was transiently expressed in newly generated oligodendrocytes.

To further confirm these findings and to test whether pS6RP expression in oligodendroglia shifts to much later developmental stages in CNS regions that myelinate significantly later than the spinal cord white matter, we examined and quantified pS6RP expression in cells of the oligodendroglial lineage in the mouse corpus callosum from PND7 to PND45 (Supplementary Fig. 1). Indeed, myelination starts in the corpus callosum at around PND10, while in the spinal cord, myelination onsets at around PND0–PND3.^27,28^ Co-immunolabelling for pS6RP and Sox10 in the *genu* of corpus callosum revealed a peak of pS6RP expression in Sox10^+^ oligodendroglia at PND14 and a subsequent decrease at PND21 and PND45 (Supplementary Fig. 1A–D, Q and R). Similar to the spinal cord, pS6RP expression in the corpus callosum was also transient and detected in a subset of CC1^+^BCAS1^+^ early myelinating oligodendrocytes (Supplementary Fig. 1E–H, I–L, S, T and U). At PND14, nearly 31 ± 9.8% of CC1^+^ oligodendrocytes were co-labelled for pS6RP and BCAS1, and the peak of pS6RP expression coincided with the occurrence of the first MOG^+^ myelin sheath segments in the corpus callosum at PND14 (Supplementary Fig. 1M–P, Q and V), suggesting that S6K activity in oligodendroglia is correlated with early myelination.

Overall, our data demonstrate that (i) pS6RP expression is timely regulated during oligodendroglial lineage progression; (ii) pS6RP is mainly detected in maturing oligodendrocytes and correlates with active myelination and (iii) pS6RP expression in oligodendrocytes is downregulated by the time developmental myelination is complete. These findings support the hypothesis that S6K activity and pS6RP are critical for oligodendrocyte maturation and/or the initiation of myelination.

### pS6RP is upregulated in differentiating oligodendrocytes following acute LPC-induced demyelination in mice

**Figure 3:**
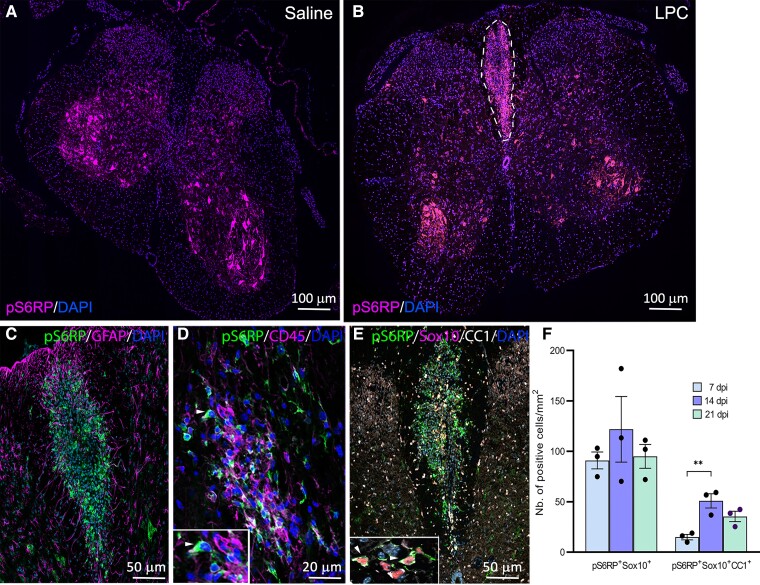
**Expression of pS6RP in LPC-induced demyelinated lesions**. (**A**) Expression of pS6RP in the saline-injected spinal cord. (**B**) pS6RP immunolabeling in LPC-induced demyelinated spinal cord at 7 dpi. pS6RP is upregulated in the LPC lesion of the dorsal funiculus (dashed lines). (**C**) Co-immunolabelling of pS6RP and GFAP. Note that GFAP^+^ astrocytes are not stained with the anti-pS6RP antibody. (**D**) Co-immunolabelling of pS6RP and CD45 reveals expression in few monocytes/macrophages (arrowhead, inset). (**E**) Triple immunostaining for pS6RP, Sox10 and CC1. Inset (**E**) illustrates the expression of pS6RP in Sox10^+^CC1^+^ oligodendrocytes in the lesion at 7 dpi (arrowheads). (**F**) Quantification of pS6RP-expressing oligodendrocytes showed a peak of pS6RP expression at 14 dpi, coinciding with the remyelination phase of the lesion. Data represent mean ± SEM; *N* = 3 independent experiments at each timepoint. ANOVA followed with *post hoc* Tukey's pairwise multiple comparison tests: **P ≤ 0.01. Scale bars: (**A** and **B**), 100 µm; (**C** and **E**), 50 µm; (**D**), 20 µm.

To determine if S6K is also active in differentiating oligodendrocytes during remyelination, we examined the expression pattern of pS6RP after focal demyelination induced by LPC injection in the adult mouse spinal cord. Interestingly, we found that pS6RP expression is highly upregulated in LPC-induced demyelinated lesions from 7 to 21 days post-injection (dpi), whilst no expression was detected in the normal white matter of saline-injected spinal cords or in normal-appearing white matter of LPC-injected spinal cords (Fig. 3A and B). Quantification of the density of pS6RP-expressing cells per lesion area revealed a peak of expression from 14 to 21 dpi, coinciding with the remyelination phase of the LPC lesion.^15,29^ To further characterize the expression profile of pS6RP in oligodendroglial cells located in demyelinated lesions, we performed triple IHC for pS6RP and oligodendroglial lineage-specific markers. We found pS6RP expression mainly in Sox10^+^CC1^+^ differentiated oligodendrocytes recruited into the lesion (Fig. 3E and F), whereas no expression of pS6RP in GFAP^+^ astrocytes was detected at all-time points post-demyelination (Fig. 3C). In addition, we observed the expression of pS6RP in few scattered CD45^+^ cells (Fig. 3D). The density of pS6RP^+^Sox10^+^ oligodendroglial cells and pS6RP^+^Sox10^+^CC1^+^ mature oligodendrocytes was significantly higher at 14 and 21 dpi relative to 7 dpi (Fig. 3F). Taken together, these data suggest a specific activation of S6K signalling in oligodendroglial cells during their differentiation into remyelinating oligodendrocytes after demyelination, similar to what we observed during developmental myelination.

### pS6RP is detected in oligodendroglial cells in mouse EAE and human MS lesions

**Figure 4:**
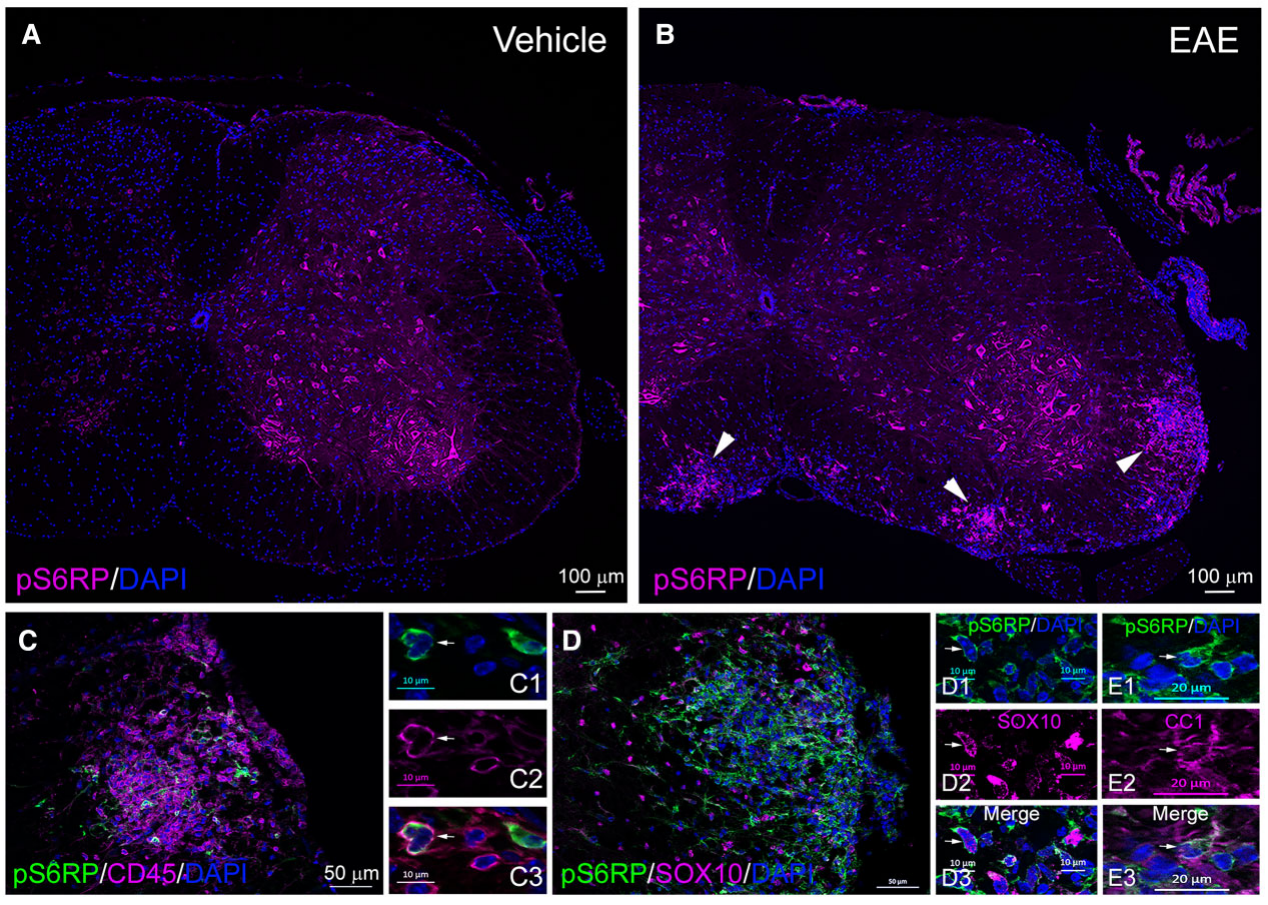
**pS6RP expression during EAE in mouse spinal cord**. (**A**) pS6RP immunolabelling in the naive adult spinal cord. Note that pS6RP is mainly detected in motor neurons and absent in white matter in the naive adult spinal cord. (**B**) pS6RP immunostaining in MOG-induced EAE lesions of the spinal cord. Inflammatory foci are indicated by arrowheads. (**C**–**E**3) pS6RP-expressing cells within EAE lesions are predominantly CD45^+^ cells (**C**, insets **C**1–**C**3) or Sox10^+^ (**D**, insets **D**1–**D**3) and CC1^+^ (insets **E**1–**E**3) oligodendrocytes. Arrows in insets indicate double-positive cells. Scale bars: (**A** and **B**), 100 µm; (**C** and **D**), 50 µm; (**C**1–**C**3, **D**1–**D**3), 10 µm; (**E**1–**E**3), 20 µm.

We next examined pS6RP expression in the EAE model of inflammatory demyelination. EAE was induced in C57BL6 mice by immunization with MOG_35–55_ peptides. Control mice were treated with CFA alone. Mice were scored daily, from 10 to 40 dpi, to assess the clinical course of the disease. Typical EAE lesions with CD45^+^ immune infiltrates were detected in dorsal and ventral white matter spinal cord tracts and correlated with the severity of the disease course. In control animals, pS6RP was only detected in spinal cord motor neurons (Fig. 4A), whilst in MOG-induced EAE mice, pS6RP expression was highly upregulated in EAE lesions (Fig. 4B). Double immunostaining for pS6RP and cell lineage-specific markers showed pS6RP expression in CD45^+^ immune cells (Fig. 4C, insets 4C1–C3) and Sox10^+^, as well as CC1^+^ oligodendrocytes (Fig. 4D, insets 4D1–D3 and 4E1–E3), whereas no expression was detectable in astrocytes (data not shown).

**Figure 5:**
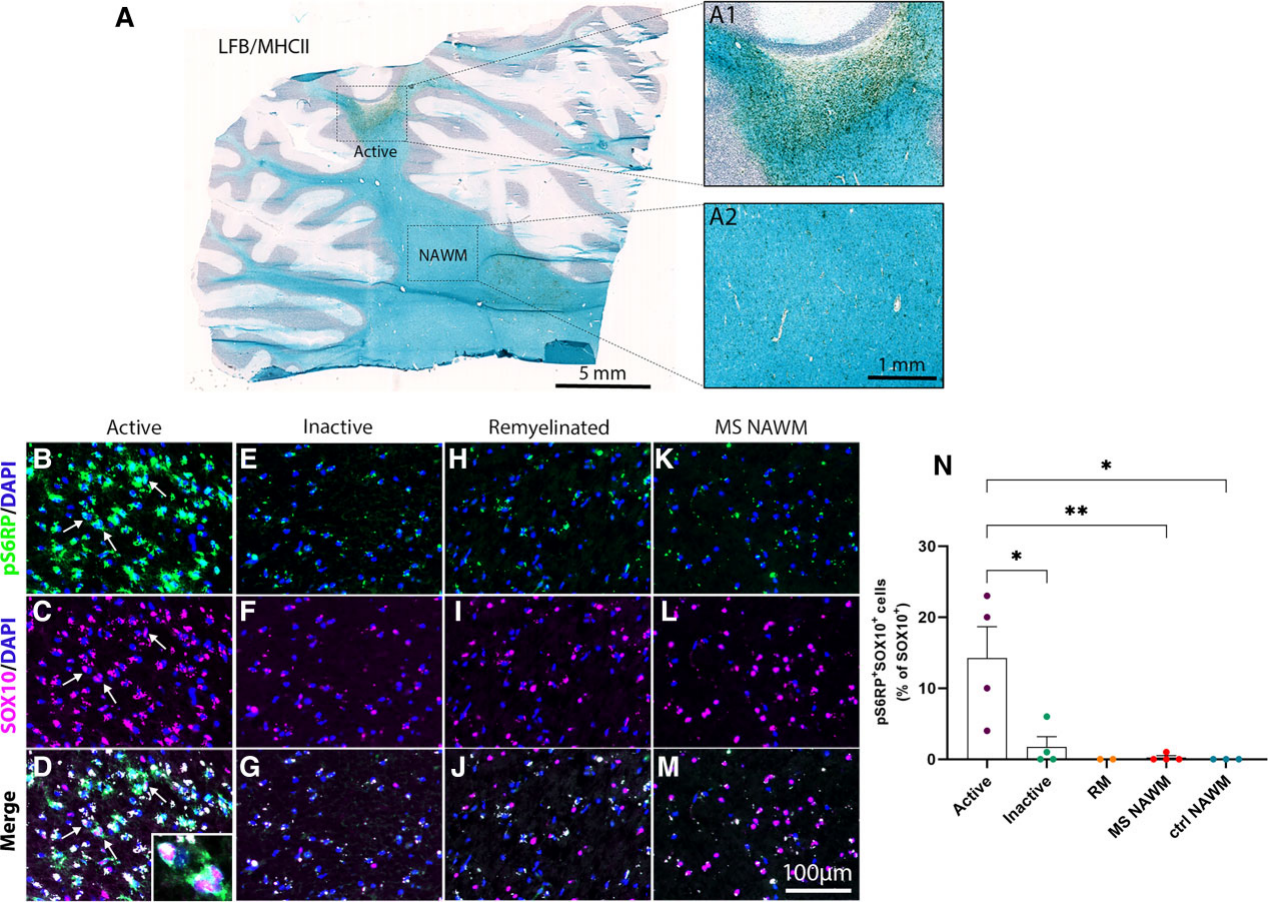
**Immunodetection of pS6RP in MS lesions**. (**A**) Luxol fast blue/MHCII staining illustrating an active MS lesion located in the cerebellar white matter. The boxed areas in panel **A** illustrate a typical active plaque filled with MHCII^+^ immune cells (**A**1) and the normal-appearing white matter (**A**2), respectively. Co-immunolabelling for pS6RP (green) and Sox10 (magenta) indicates that pS6RP is mainly detected in oligodendroglia (arrows) in active (**B**–**D**, inset in **D**) but not in chronic inactive (**E**–**G**) nor in remyelinated lesions (**H**–**J**) and normal-appearing white matter (**K**–**M**). Quantification of the percentage of Sox10^+^ oligodendroglial cells expressing pS6RP in different MS lesions subtypes and normal-appearing white matter from MS and controls cases (**N**). Data represent mean ± SEM for each MS lesion type. Active: active lesions (*n* = 3) and rims of chronic active lesions (*n* = 1); Inactive: Chronic inactive (*n* = 3) and core of chronic active (*n* = 1) lesions; RM: remyelinated lesions (*n* = 2); MS NAWM: normal-appearing white matter from MS cases (*n* = 4); Ctl NAWM: normal-appearing white matter from controls (*n* = 3). ANOVA followed with *post hoc* Tukey's pairwise multiple comparison tests: *P ≤ 0.05; **P ≤ 0.01. Panels (**B–M**) are counterstained with Dapi. Scale bars: (**A**), 5 mm; (**A**1 and **A**2), 1 mm; (**B**–**M**), 100 µm.

We then determined whether pS6RP would be detectable in oligodendroglia in MS post-mortem brain tissue. For this study, we used snap-frozen cerebellar tissue sections from eight MS cases and three controls with non-neurological diseases (Supplementary Table 1). MS lesions were identified using Luxol fast blue/MHCII staining (Fig. 5A) and classified as active, chronic active, chronic silent and shadow plaques.^16,30^ Interestingly, pS6RP^+^Sox10^+^ oligodendroglial cells were mainly detected in active lesions (Fig. 5A and B–D) and in rims of chronic active lesions (not shown) but were almost absent in chronic inactive lesions (Fig. 5E–G), in remyelinated lesions (Fig. 5H–J) and in the normal-appearing white matter from MS (Fig. 5K–M) and control cases. Quantification of the percentage Sox10^+^pS6RP^+^ cells over the total number of Sox10^+^ oligodendroglia in different MS lesion types revealed a peak of expression in active lesions, as compared with chronic silent lesions, shadow plaques and normal-appearing white matter (Fig. 5N), suggesting that pS6RP labelled a transient stage of the oligodendroglial lineage cells in human.

### Inhibiting S6K1 activity prevents oligodendrocyte differentiation *in vitro*

pS6RP is a primary target of the S6Ks and a read-out of S6K activity. Both S6K1 and S6K2 phosphorylate S6RP; however, S6K1 is the predominant regulator of cell growth in many tissues.^1,3^ Thus, we hypothesize that S6K1 is active in differentiating oligodendrocytes as they extend processes and initiate myelination. This hypothesis is supported by RNAseq data indicating that S6K1 (*Rps6kb1*) and S6K2 (*Rps6kb2*) transcripts are expressed in cells of the oligodendroglial lineage (see http://linnarssonlab.org/oligodendrocytes/).^31,32^ However, the mRNA expression level of S6K1 is around 3-fold higher in OPCs and myelinating oligodendrocytes than that of S6K2. Therefore, we assume that S6RP is the main target of S6K1 in oligodendroglia.

To analyse S6K1 function in oligodendrocyte differentiation, we tested the effect of the inhibitor PF-4708671,^33^ which has 400-fold greater selectivity for S6K1 than S6K2 in a rat OPC line (CG4) undergoing differentiation *in vitro*. Similar to primary rat OPCs, the CG4 cell line can be maintained as immature PDGFRα^+^O4^−^ OPCs in the proliferation medium and can be induced to differentiate into O4^+^ GalC^+^ differentiated oligodendrocytes upon mitogen withdrawal.^21^ Based on prior studies indicating PF-4708671 has specificity for S6K1 in the range of 1–10 µM,^33^ we initially treated CG4 cells with the inhibitor at 1 µM during 3 days of differentiation. This dose was sufficient to inhibit CG4 cell differentiation, as indicated by GalC immunolabelling (Ctl = 10.9 ± 1.52%; PF = 0.55 ± 0.3%; Supplementary Fig. 2A, B and F) and to inhibit the appearance of pS6RP^+^ cells in the cultures (Ctl = 52.56 ± 3.6%; PF = 5.26 ± 1.56%; Supplementary Fig. 2C–E).

**Figure 6:**
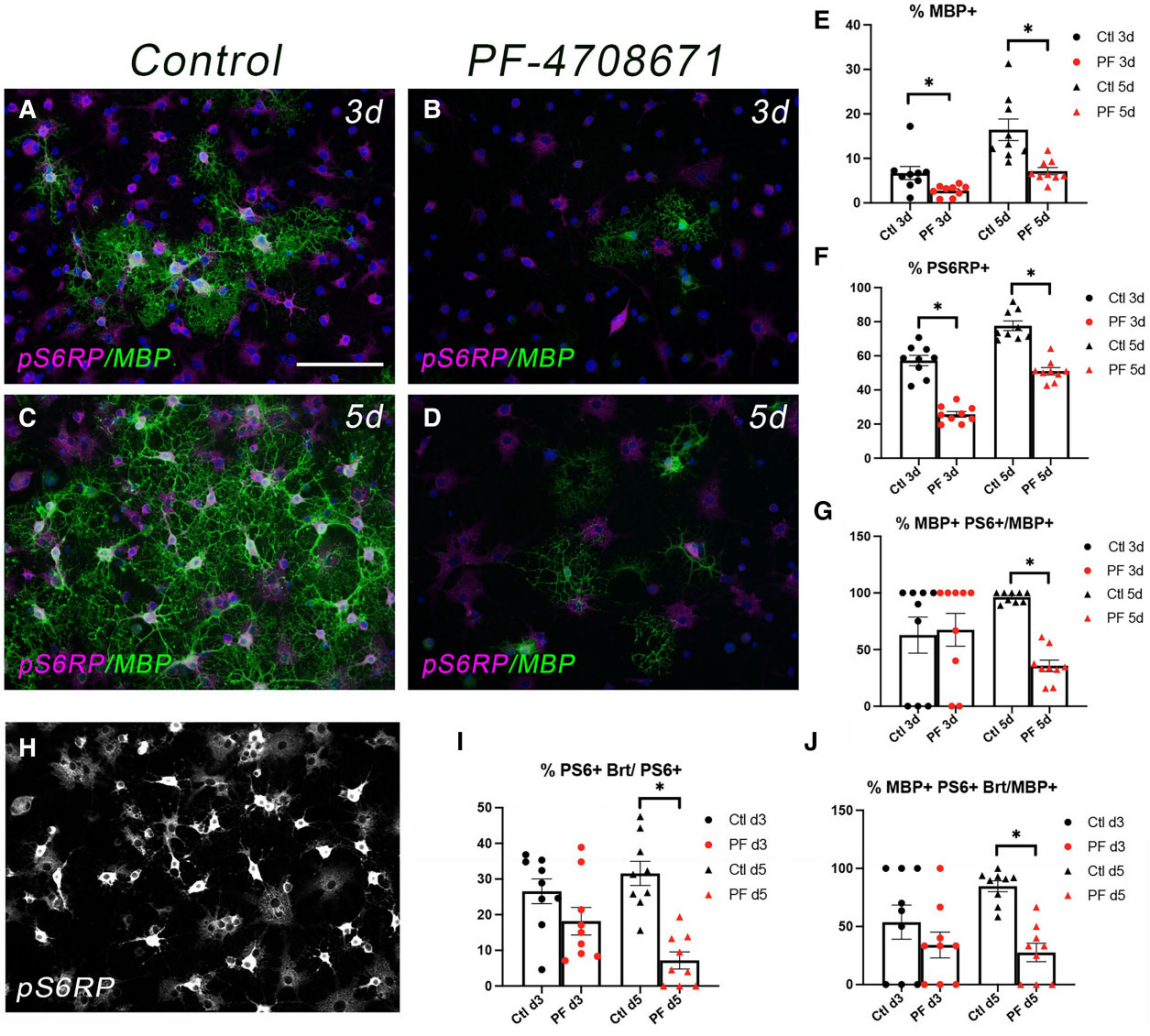
**p70S6K1 activity correlates with and regulates the onset of MBP expression in primary rat OPCs *in vitro***. (**A**–**D**) Immunostaining for MBP (green) and pS6RP (pink) in primary rat OPCs after 3 days (**A** and **B**) or 5 days (**C** and **D**) of differentiation in the absence (**A** and **C**) or presence (**B** and **D**) of PF-4708671 (10 µM). Nuclei are stained with Dapi (blue). (**E**–**G**) Graphs indicating the percentage of cells at each timepoint that were MBP^+^ (**E**), pS6RP^+^ (**F**) and MBP^+^PS6RP^+^ (**G**). (**H**) Immunostaining for pS6RP after 5 days in control differentiation media (same image as panel **C**) showing both bright (brt) and low or diffuse staining for pS6RP. (**I**) Graph indicating percentage of PS6RP^+^ cells at each timepoint that was bright. (**J**) Graph indicating percentage of MBP^+^ cells at each timepoint that were also PS6RP^+^. An unpaired two-tailed *t*-test with Welch's correction if variances are unequal: *P < 0.0001 for all graphs with the exception of panel **E** (3 days, *P* = 0.0272 and 5 days, *P* = 0.0049). Scale bars (**A**–**D** and **H**), 100 µm.

We next compared the effect of the S6K1 inhibitor with the mTOR inhibitor, rapamycin, on the expression of pS6RP and MBP by western blot analyses of cell lysates from primary rat OPCs undergoing differentiation (Supplementary Fig. 3). Treatment of cells with 10 µM PF-4708671 inhibited pS6RP expression after 24 h (Supplementary Fig. 3A) and completely inhibited MBP protein expression after 72 h of differentiation (Supplementary Fig. 3B). The reductions in pS6RP and MBP expression with the S6K1 inhibitor were equivalent to the effect of the mTOR inhibitor rapamycin (Supplementary Fig. 3A and B) at doses we used in prior studies to inhibit OPC differentiation.^9^ Immunostaining of primary rat oligodendroglia undergoing differentiation further revealed that the S6K1 inhibitor prevented the differentiation of the cells into MBP^+^ oligodendrocytes (Fig. 6).

To further define the function of S6K1 in oligodendrocyte differentiation, we treated primary rat OPCs with 10 µM PF-4708671 (PF) and analysed the cellular expression of MBP and pS6RP after 3 and 5 days of differentiation (Fig. 6). Inhibiting S6K1 reduced the percentage of MBP^+^ and pS6RP^+^ cells at both 3 and 5 days of differentiation (Fig. 6A–F). At 3 days of differentiation in control cultures, 6.7% of the cells were MBP^+^ and 57% were pS6RP^+^ (Fig. 6E and F). PF treatment reduced the percentage of MBP^+^ cells to 2.7% (*P* = 0.0272) and the percentage of pS6RP^+^ cells to 25.7% (*P* < 0.0001; Fig. 6E and F). Interestingly, approximately the same percentage of MBP^+^ cells were also pS6RP^+^ in both control and PF-treated cultures at 3 days (Ctl = 62.8%; PF = 67.4%, Fig. 6G) even though the percentage of MBP^+^ cells was low at this early timepoint. These data support the conclusion that initiation of MBP expression in differentiating OPCs is dependent, at least in part, on S6K1 activity. The percentage of MBP^+^ and pS6RP^+^ cells continued to be negatively impacted by inhibiting S6K1 after 5 days of differentiation (Fig. 6C–F). The percentage of MBP^+^ cells in control cultures increased to 16.4% but only to 7.2% in PF-treated cultures (*P* = 0.0049; Fig. 6E), whereas the percentage of pS6RP^+^ cells was 77.6% and 51% in control versus PF-treatments, respectively (*P* < 0.0001; Fig. 6F). However, unlike d3, the percentage of MBP^+^ cells that was also pS6RP^+^ was now significantly reduced by PF treatment at (Ctl = 96.3%, PF = 35.7%, *P* < 0.0001; Fig. 6G).

Analyses of pS6RP expression during OPC differentiation *in vitro* revealed both high and low expressing populations in control conditions (Fig. 6H). The percentage of pS6RP^+^ cells that were ‘bright’ at d3 was not significantly different between control versus PF-treated cultures (Ctl = 26.6%, PF = 18.2%). However, by d5 of differentiation, only 7.2% of the pS6RP^+^ cells were bright in the PF-treated cultures compared with 31.6% of the pS6RP^+^ cells in the control cultures (*P* < 0.0001; Fig. 6I). Thus, 84.7% of the MBP^+^ cells were also S6RP^+^ bright in control conditions at this time compared with only 27.6% of the PF-treated cells (*P* < 0.0001; Fig. 6J). Taken together with the data shown in Fig. 6G, these data indicate that the new MBP^+^ cells in the PF-treated cultures at d5 utilized a pathway distinct from S6K1 to initiate MBP expression. However, our results support a critical role for S6K1 function in oligodendrocyte differentiation and myelination.

**Figure 7:**
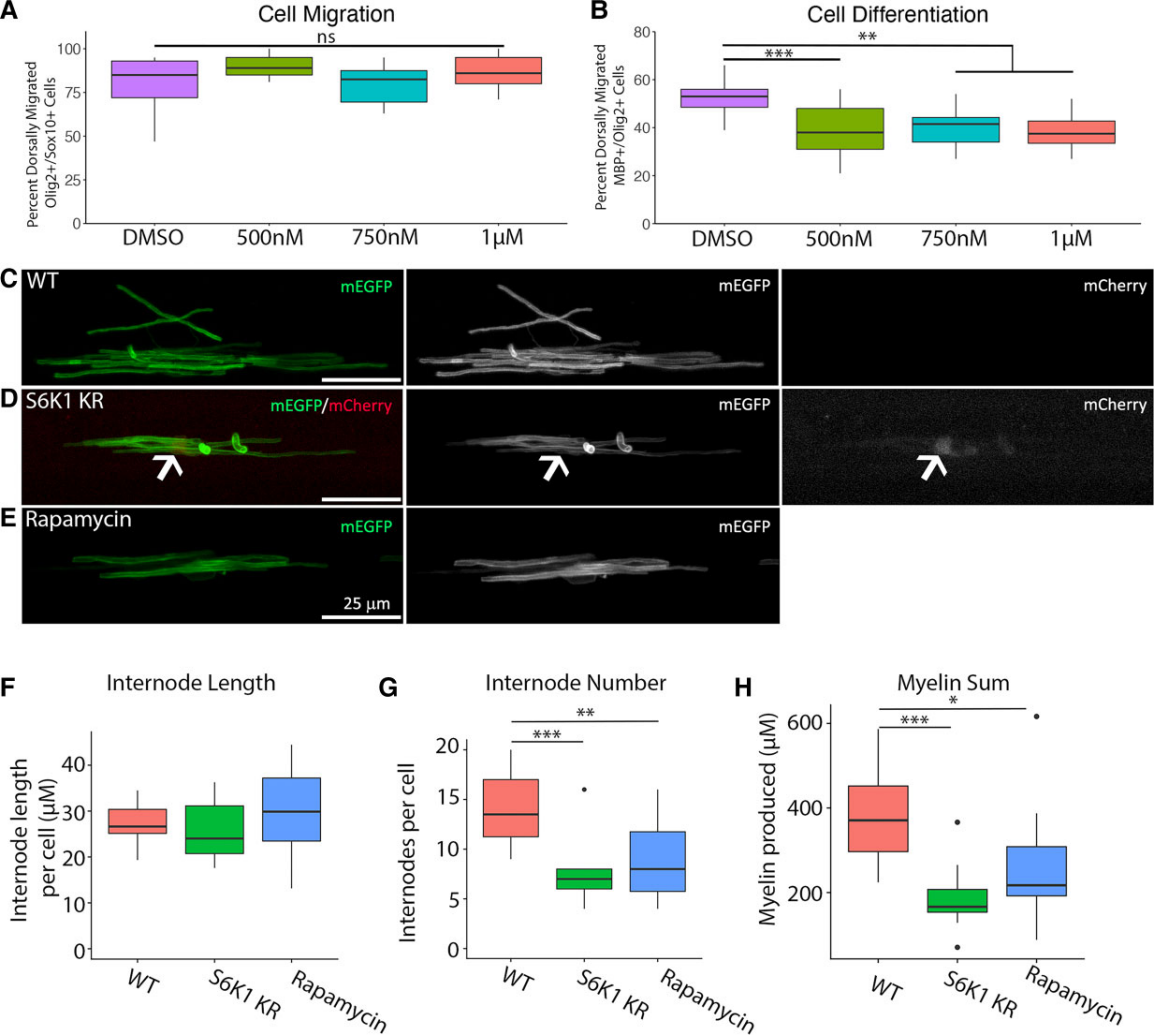
**Inhibiting S6K1 kinase activity in oligodendrocytes decreases myelin production *in vivo***. (**A**) The per cent of dorsally migrated Olig2^+^ cells that were Olig2^+^/Sox10^+^ in the ventral spinal cord of zebrafish treated with p70S6K1 inhibitor PF-4708671 from 2 to 3 dpf. ANOVA *F*(3,33) = 1.971, ns, *P* = 0.1374. (**B**) The per cent of all Olig2^+^ dorsally migrated cells that were Mbp^+^/Olig2^+^ treated with p70S6K1 inhibitor PF-4708671 from 2 to 4 dpf. ANOVA *F*(3,51) = 7.275, *P* = 0.004, Dunnett's multiple comparisons test adjusted *P*-value **P = 0.0012–0.0032, ***P = 0.0006, *n* = 9–15 fish per treatment group. (**C**–**E**) Representative images of myelin internodes. Zebrafish embryos were injected with pMBP:mEGFP along with a second plasmid to manipulate S6K1 function, labelled with cytoplasmic RFP or treated with the mTOR inhibitor rapamycin from 2 to 4 dpf. All images were taken in the ventral spinal cord above the yolk sac extension. (**C**) WT cell expressing pMBP:mEGFP to label internodes. (**D**) A cell expressing both pMBP:mEGFP to label internodes and pMYRF:S6K1-KR to inhibit S6K1 function, labelled with red fluorescent protein (arrowhead). (**E**) pMBP:mEGFP^+^ cell in rapamycin-treated zebrafish. (**F**) Quantification of myelin internode length averaged per cell. Internode length: ANOVA, *F*(2,24) = 2.731, ns, *P* = 0.0854. (**G**) Quantification of myelin internode number averaged per cell. ANOVA, *F*(2,24) = 10.25, *P* = 0.0006; with Tukey's multiple comparisons test adjusted *P*-value: **P = 0.0024, ***P = 0.0009, (**H**) Sum of myelin produced per cell. ANOVA, *F*(2,24) = 8.259, *P* = 0.0019; with Tukey's multiple comparisons test adjusted *P*-value: *P = 0.0026, **P = 0.014. All images were analysed in 3D using IMARIS software. *n* = 10–14 cells per condition (one cell per fish). Scale bars (**C**–**E**), 25 µm.

### Inhibiting S6K1 reduced the number of myelin internodes in zebrafish *in vivo*

To further determine if S6K1 activity regulates OPC migration and differentiation *in vivo*, we inhibited S6K1 in zebrafish larvae. Thus, to examine whether S6K1 activity was required during OPC migration in zebrafish, we treated larval zebrafish with increasing concentrations of the S6K1 inhibitor, PF4708671 (Fig. 7A and B). Treatment during OPC migration, from 2 to 3 dpf, had no impact on migration of Sox10^+^Olig2^+^ progenitor cells from the ventral to the dorsal spinal cord (Fig. 7A). However, consistent with our *in vitro* data, extending the S6K1 inhibitor treatment to include oligodendrocyte differentiation (2–4 dpf) led to a significant reduction in differentiated MBP^+^ cells among the Olig2^+^ population in the spinal cord (Fig. 7B).

Next, to determine if S6K1 activity regulates myelin production *in vivo*, we investigated myelination in the zebrafish spinal cord. A kinase-dead version of S6K1,^24^ driven by an oligodendrocyte-specific promotor, was injected at the one-cell stage to inhibit S6K1 function in zebrafish oligodendrocytes. This plasmid also contained a 2A peptide cleavable NLS-mCherry, which labels the nuclei of cells expressing the S6K1-KR with mCherry without tagging the protein (pEXPR-myrf:S6K1-KR-2AnlsmCherry, henceforth referred to as pMyrf:S6K1-KR). By co-injecting the S6K1 mutant with a plasmid to label myelin production (pEXPR-MBP:mEGFP, henceforth referred to as pMBP:mEGFP), we were able to quantify the amount of myelin produced by individual oligodendrocytes in WT or S6K1-KR^+^ cells (Fig. 7C and D). This mosaic expression allowed us to quantify several aspects of myelination, including the number of myelin internodes produced per cell and the length of individual internodes for an individual cell. Cells expressing the kinase-dead S6K1 plasmid had fewer myelin internodes per cell, but there was no change in the myelin internode length (Fig. 7F–H). Thus, inhibiting S6K1 in a cell-specific manner decreased total myelin production per cell, primarily by reducing the number of total internodes. These results demonstrate that S6K1 positively regulates myelin production.

To determine if inhibiting mTOR *in vivo* led to a similar phenotype as S6K1 inhibition, we injected embryos at the one-cell stage with a plasmid to label myelin internodes, pMBP:mEGFP, and treated zebrafish with the mTOR inhibitor rapamycin or DMSO vehicle control from 2 to 4 dpf (Fig. 7C and E). Inhibiting mTOR led to a decrease in myelin internodes produced per cell without altering myelin internode length (Fig. 7F–H). These results demonstrate that globally inhibiting mTOR *in vivo* leads to a decrease in myelin production, similar to the oligodendrocyte-specific S6K1 inhibition. These data suggest that mTOR and S6K1 positively regulate myelin production.

## Discussion

Although mTOR is now well known as a regulator of oligodendrocyte differentiation and myelination,^6–13^ the specific downstream pathways mediating these actions have not been well defined. Here, we demonstrate that the mTORC1/S6K pathway is activated transiently in oligodendrocytes during developmental myelination and remyelination in mice, as well as in a subset of oligodendrocytes in active MS lesions. We also show that S6K1 mediates essential aspects of myelin production in both rodent and zebrafish oligodendrocytes.

Expression of pS6RP, a primary target of the S6Ks, revealed that S6K activity correlates with the onset of myelination during mouse spinal cord and corpus callosum development. Interestingly, in these CNS regions with a very distinct myelination time course, the peak of pS6RP expression was detected in a subset of CC1^+^BCAS1^+^ early myelinating oligodendrocytes and highly correlated with the occurrence of the first myelin internodes. *In vitro*, the high percentage of MBP^+^ cells that were also pS6RP^+^, even in early differentiation stages when the overall percentage of MBP^+^ cells was low, strongly suggests that the activity of S6K is important for initiation of myelination. The rapid downregulation of pS6RP in the mouse spinal cord between PND10 and PND15, and in the corpus callosum from PND21 to PND45, was particularly striking and supports the hypothesis not only that activation of S6K is necessary for the onset of myelination but also that its inactivation is also important.^34^ Similar to our findings in oligodendrocytes, the activity of S6K as determined by pS6RP expression, is high in Schwann cells both *in vitro* and during peripheral nerve development prior to myelination and then decreases at the onset of myelination.^34,35^ That activity of S6K that needs to decrease for myelination to proceed properly is supported by the transient peak of activity observed in both the CNS and PNS myelin-producing cells at the onset of myelination as well as the studies in Schwann cells demonstrating that hyper-activation of mTORC1 in Schwann cells through deletion of *Tsc1*, the upstream mTORC1 inhibitor, results in impaired Schwann cell differentiation and a delay in the onset of myelination in peripheral nerves.^36^ The effect of *Tsc1* deletion on Schwann cell differentiation in this study was reflected in the reduced expression of Krox20, an essential transcriptional regulator of Schwann cell differentiation. Importantly, expression of Krox20 in Schwann cells lacking *Tsc1* was partially rescued by pharmacological inhibition of S6K as was their ability to myelinate dorsal root ganglion neurons *in vitro*.^36^ Taken together with our findings, these data support the conclusion that the timing of mTORC1/S6K activation is critical for both CNS and PNS myelination.

pS6RP expression is a reliable read-out of S6K activity but does not distinguish between the activity of S6K1 or S6K2. S6K1 and S6K2 both are activated by mTORC1 phosphorylation on Thr389 and are the main kinases that phosphorylate S6RP. However, S6K1 and S6K2 are differentially regulated, and the currently known substrates of S6K1 and S6K2 are mostly distinct with the exception of S6RP.^1,37^ Like S6K1, S6K2 is phosphorylated by mTORC1 and PDK1 but is regulated distinctly by MEK/ERK, interleukin3 and protein kinase C. S6K1 is a known effector of several other proteins including mTOR, rictor, neurabin, PDK1, PLD1, Rac1/Cdc42 and SIRT1/SIRT2. S6K1 also exerts negative feedback to suppress Akt activation through inhibiting insulin receptor substrate 1 and PDK1 phosphorylation at Thr308, as well as the mTORC2/rictor phosphorylation site at Ser473. Loss of S6K1 systemically in mice results in increased sensitivity to insulin signalling through augmenting Akt activation.^38^ Consistent with these findings, pharmacological inhibition of S6K1, systemically using the specific inhibitor PF4708671,^33^ resulted in increased Akt phosphorylation at both Thr308 and Ser473 in heart tissue.^39^ In contrast to these findings and in support of diverse functions of S6K, Figlia *et al*.^36^ showed that the effect of hyperactive mTORC1 in early Schwann cell differentiation was independent of activating PI3K/Akt.

Our functional analyses using the specific S6K1 inhibitor in differentiating rat oligodendrocytes *in vitro* or in zebrafish *in vivo* and through expressing an S6K1 dominant-negative construct in differentiating zebrafish oligodendrocytes reveals an essential function for S6K1 in oligodendrocyte differentiation and myelination. These data suggest that S6K2 is not highly active in differentiating oligodendrocytes at this time, which is also supported by our data showing that the S6K1 inhibitor almost completely blocks phosphorylation of S6RP, a substrate for both S6K1 and S6K2, in differentiating OPCs *in vitro* (Supplementary Fig. 2A). The zebrafish data showing fewer myelin internodes per cell with reduced S6K1 activity is also consistent with the well-established function of S6K1 in regulating cell size.^1,3^ It is likely that S6K1 has distinct targets that regulate early myelination. Interestingly, a recent study identified additional cdk5-dependent serine phosphorylation sites in the C-terminus of S6K1 that are necessary for S6K1 phosphorylation of specific substrates in insulin-stimulated adipocytes but not for S6RP phosphorylation.^40^ Potentially relevant to the myelination programme in oligodendrocytes, some of the identified substrates regulate lipid metabolism.

Our data support the hypothesis that mTORC1 has a major function in regulating myelin production and myelination. This is consistent with prior data from our laboratories demonstrating that deletion of raptor in developing OPCs driven by the CNP-Cre promoter phenocopies deficits in spinal cord myelination observed from deletion of mTOR by CNP-Cre expression. Here, we also show that S6K1 inhibition in zebrafish oligodendrocytes similarly reduces myelin production per oligodendrocyte as seen with mTOR inhibition with rapamycin.

## Supplementary Material

fcac025_Supplementary_Data

## Abbreviations

APC/CC1 =: adenomatous polyposis coli
BCAS1 =: breast carcinoma amplified sequence 1
DAPI =: 4′,6-diamidino-2-phenylindole
DMSO =: dimethyl sulfoxide
Dpf =: days post-fertilization
EAE =: experimental autoimmune encephalomyelitis
ERK =: extracellular signal-regulated kinases
FGF =: fibroblast growth factor
GalC =: galactocerebrosidase
GFAP =: glial fibrillary acidic protein
HLA =: human leucocyte antigen
Hpf =: hour post-fertilization
LPC =: lysophosphatidylcholine
MBP =: myelin basic protein
MEK =: mitogen-activated protein kinase kinase
MHCII =: major histocompatibility complex class II
MOG =: myelin oligodendrocyte glycoprotein
MS =: multiple sclerosis
mTOR =: mammalian target of rapamycin
mTORC1 =: mammalian target of rapamycin complex 1
mTORC2 =: mammalian target of rapamycin complex 2
OPC =: oligodendrocyte progenitor cell
PDGFRα =: platelet-derived growth factor receptor alpha
PDK1 =: 3-phosphoinositide-dependent protein kinase-1
PI3K/Akt =: phosphoinositide 3-kinase/protein kinase B
PLD1 =: phospholipase D1
pS6RP =: phosphorylated S6 ribosomal protein
Rac1/Cdc42 =: Ras-related C3 botulinum toxin substrate 1/cell division control protein 42 homologue
S6K1 =: p70 ribosomal S6 kinase 1
S6K2 =: p70 ribosomal S6 kinase 2
S6RP =: S6 ribosomal protein
SIRT1/SIRT2 =: sirtuin 1/sirtuin 2
Sox10 =: SRY-related HMG-box 10
Tsc1 =: tuberous sclerosis 1
