# Supplementary material for: p70S6 kinase regulates oligodendrocyte differentiation and is active in remyelinating lesions
Source: Brain Commun. 2022 Feb 12;4(1):fcac025. doi: 10.1093/braincomms/fcac025 (PMC8864467; doi:10.1093/braincomms/fcac025)
Supplement: fcac025_Supplementary_Data [file fcac025_supplementary_data.pdf]

Supplementary Material

Supplementary Figure 1

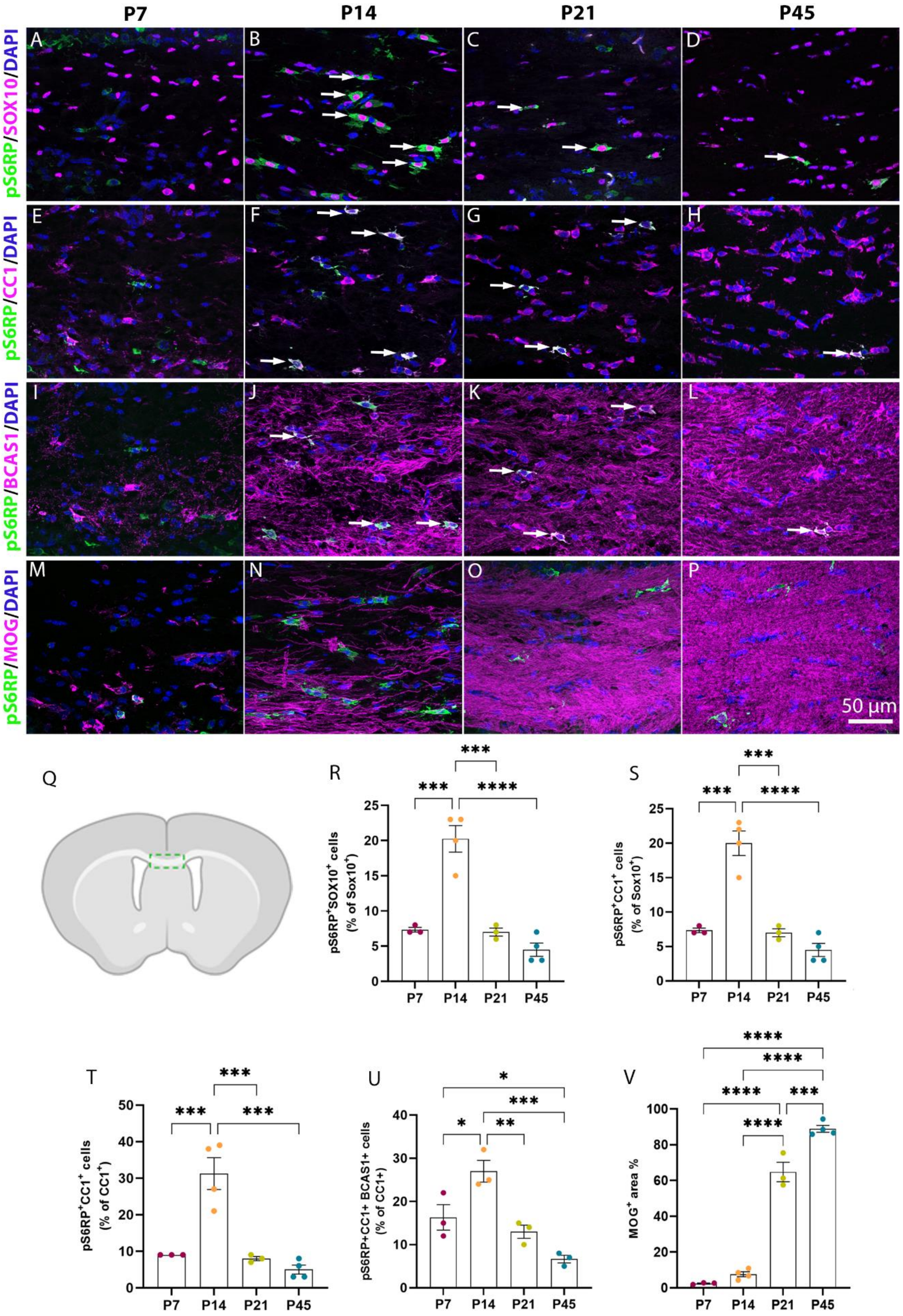

Supplementary Figure 1. Expression profile of pS6RP in the developing mouse corpus callosum.

pS6RP co-immunolabeling with Sox10 (**A-D**), CC1 (**E-H**), BCAS1 (**I-L**) or MOG in the *genu* of the mouse corpus callosum (dashed area, **Q**), at PND7 (**A**), PND14 (**B**), PND21 (**C**) and PND45 (**D**). Quantification of the percentages of Sox10+ oligodendroglia expressing pS6RP (**R**) or pS6RP and CC1 (**S**), at different developmental stages. Percentage of CC1+ mature oligodendrocytes stained for pS6RP (**T**), pS6RP and BCAS1 (**U**), at several developmental time points. Quantification of MOG+ myelinated area in the *genu* of the corpus callosum. Note that pS6RP expression is detected in few CC1+BCAS1+ early myelinating oligodendrocytes. Arrows indicate double positive cells. As in the spinal cord, not all CC1+ oligodendrocytes express pS6RP, suggesting a transient expression in maturing oligodendrocytes. Data represent mean $\pm$ SEM of 3-4 independent experiments per developmental stage. ANOVA with post-hoc Tukey's multiple comparison tests: *$p \leq 0.05$, **$p \leq 0.01$, ***$p \leq 0.001$, ****$p \leq 0.0001$. Scale bar: 50 μm.

Supplementary Figure 2

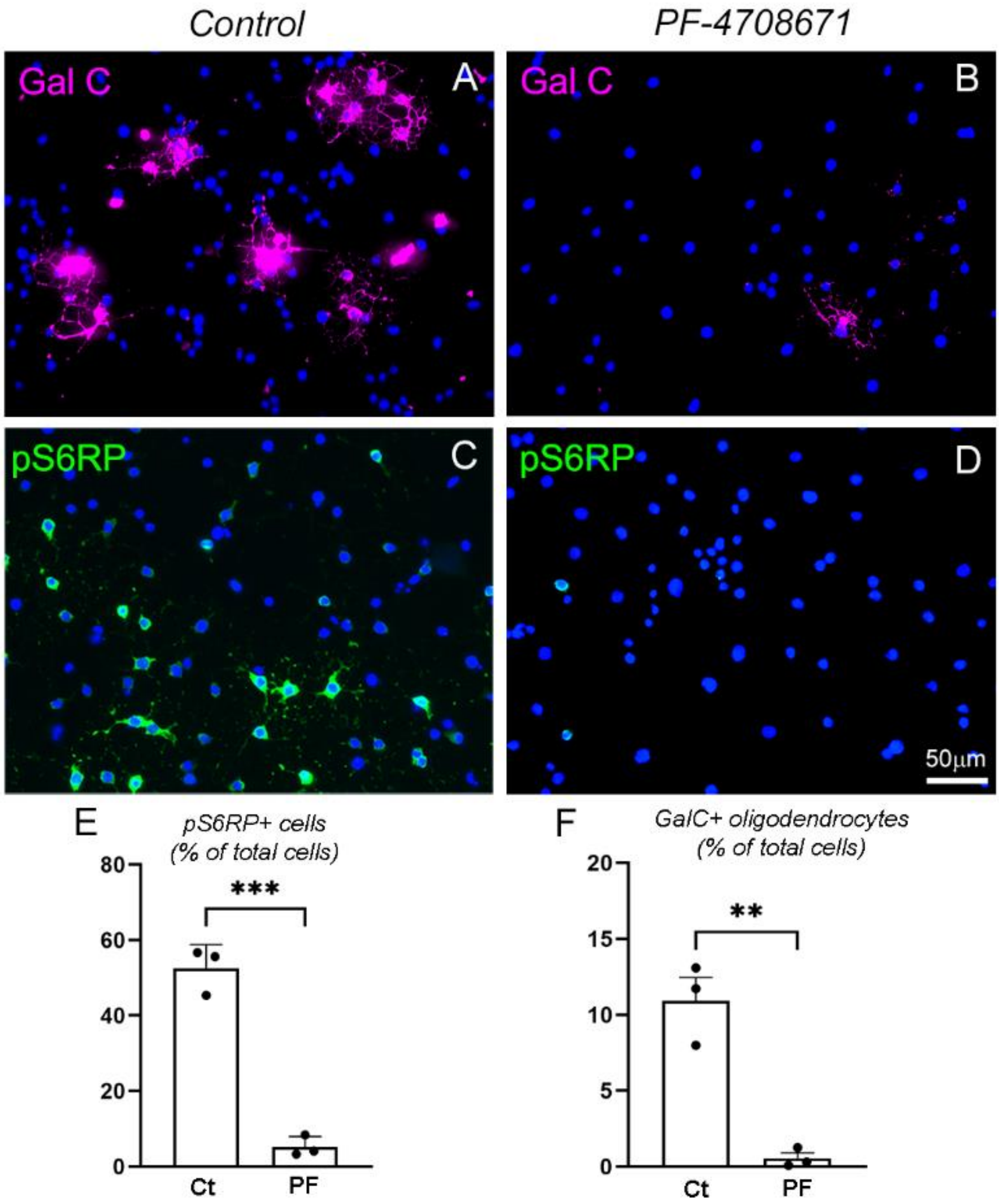

Supplementary Figure 2. The p70S6K1 specific inhibitor, PF-4708671, prevents the differentiation of CG4 cells into oligodendrocytes, *in vitro*.

(A-D) Immunostaining for GalC (A-B) and pS6RP (C-D) in CG4 cell cultures, after 3 days in differentiation media in the absence (A,C) or presence (B,D) of PF-4708671 (1 µM). Nuclei were stained with Dapi. (E-F) Graphs indicating the percentage of pS6RP+ (E) and GalC+ cells (F) in CG4 cell cultures, treated and untreated with PF-4708671 inhibitor. Data represent mean ± SEM of 3 independent experiments. Unpaired two-tailed t-tests: $^{**}p \leq 0.01$, $^{***}p \leq 0.001$. Scale bar (A-D), 50 µm.

Supplementary Figure 3

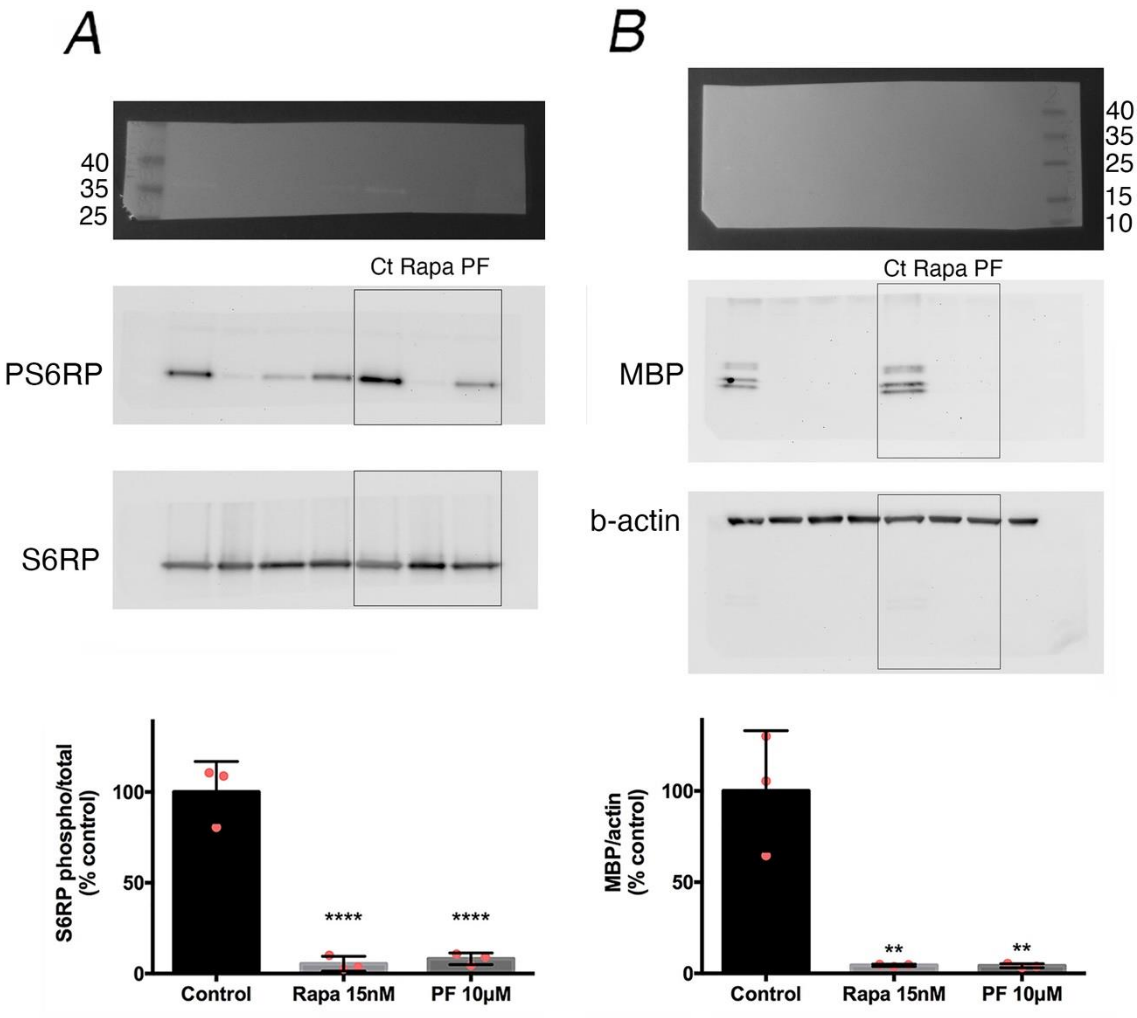

Supplementary Figure 3. The p70S6K1 specific inhibitor, PF-4708671, and the mTOR inhibitor rapamycin equally inhibit MBP expression in primary rat OPCs *in vitro*.

Representative Western blot and quantification of pS6RP/total S6RP (A) and MBP (B) expression in primary rat OPCs after 24 hr (A) or 72 hr (B) in differentiation media alone plus DMSO vehicle (Ct) or in the presence of the mTOR inhibitor rapamycin (Rapa; 15 nM), or the S6K1 inhibitor, PF-4708671 (10 µM); n=3 independent cell preparations. MBP expression was normalized to expression of beta-actin. Preview images of the membrane blots with ladder and corresponding uncropped western blot images are shown for pS6RP and total S6RP (t-S6RP, A) and for MBP and actin (B). Molecular ladder sizes (kDa) are indicated in top preview panels (Spectra™ Multicolor Broad Range Protein Ladder, Thermo Fisher Cat# 26623). ANOVA followed by Dunnett's multiple comparison tests. A: **** $F(2,6)=84.17$, $p<0.0001$; B: ** $F(2,6)=25.16$, $p=0.0012$.

Supplementary Table 1. Clinical data of the multiple sclerosis cases and lesion sub-types used for pS6RP expression

MS cases	Age	Sexe	PMD	Disease duration	Disease course	Lesions analyzed			
						Active	Chronic active	Chronic inactive	Shadow plaques
MS74CLB9	64	F	7	36	SP	**1**			
MS76CLB2	49	F	31	18	SP				**1**
MS79CLA6	49	F	7	23	SP			**1**	
MS94_CLA6	42	F	11	6	PP	**1**			
MS100CLC2	46	M	7	8	SP			**1**	
MS121CLC5 & MS121CLB4	49	F	24	14	PR	**1**		**1**	
MS122CLA6	44	M	16	>10	SP		**1**		
MS126CLA3	75	M	22	32	SP				**1**
**Controls**									
C26CLA5	79	F	18	Cardiac failure					
C22CLA4	69	F	33	Lung Cancer					
C15CLF2	82	M	21	Unknown					

Eight MS and 3 control cases were used to study pS6RP expression. MS cases include primary progressive (PP, n=1), secondary progressive (SP, n=6) and progressive relapsing (PR, n=1) disease courses. For MS cases, the mean age is 52.25 years and the mean duration of the disease is 18.4 years. PMD: post-mortem delay.
